# Preparation of PP-g-(AA-MAH) Fibers Using Suspension Grafting and Melt-Blown Spinning and its Adsorption for Aniline

**DOI:** 10.3390/polym12092157

**Published:** 2020-09-22

**Authors:** Zhouyang Lian, Yiyang Xu, Jie Zuo, Hui Qian, Zhengwei Luo, Wuji Wei

**Affiliations:** School of Environmental Science and Engineering, Nanjing Tech University, Nanjing 211816, China; YI_AMARIS@163.COM (Y.X.); zuojieyx@163.com (J.Z.); qianhui@fiberglasschina.com (H.Q.); luozw1989@163.com (Z.L.); wjwei@njtech.edu.cn (W.W.)

**Keywords:** polypropylene, suspension grafting, melt-blown spinning, adsorption, aniline

## Abstract

This paper uses polypropylene (PP) as the matrix and acrylic acid (AA) and maleic anhydride (MAH) as functional monomers to prepare PP-g-(AA-MAH) fibers by suspension grafting and melt-blown spinning technology that are easy to industrially scale-up. The fibers can be used to adsorb aniline. Results showed that the grafting ratio reached the maximum of 12.47%. The corresponding optimal conditions were grafting time of 3 h, AA: MAH = 0.75, total monomer content of 55%, benzoyl peroxide 1.4%, xylene concentration of 6 mL/g PP, and deionized water content of 8 mL/g PP. Owing to its good fluidity and thermal stability, the product of suspension grafting can be used for melt-blown spinning. Infrared spectroscopic and nuclear magnetic resonance spectroscopic analyses indicated that AA and MAH were successfully grafted onto PP fibers. After grafting, the hydrophilicity of PP-g-(AA-MAH) fiber increased. Therefore, it had higher absorptivity for aniline and the adsorption capacity could reach 42.2 mg/g at 45 min and pH = 7. Moreover, the PP-g-(AA-MAH) fibers showed good regeneration performance.

## 1. Introduction

Water discharged from industrial activities, if left untreated or not treated to the proper standard, inevitably causes water pollution, which is one of the major environmental issues of the day [1,2]. Aniline is one of the common organic compounds used as an intermediate in chemical industry, and hence its market demand has risen sharply [3,4,5]. This provides more channels for aniline to enter water bodies, thus causing environmental pollution. Since aniline does not readily undergo degradation, it accumulates in the environment. Moreover, being a carcinogenic, teratogenic, mutagenic, and highly toxic substance, aniline has a severely high risk of causing potential damage to the environment and organisms [6,7,8]. Therefore, aniline has been included in the list of preferentially controlled pollutants by Minister of Ecology and Environment of China, the United States Environmental Protection Agency, European Chemicals Agency, and other agencies [9,10,11].

At present, treatment methods for aniline-containing wastewaters mainly include advanced oxidation [12,13], membrane separation [14,15], extraction [16,17], biological methods [18], and adsorption methods [19,20,21,22]. Among them, the adsorption method that uses the interactions between adsorbent and adsorbate to remove pollutants from wastewater has attracted the attention of researchers, because of its simple operation, fast processing, and resourceful recovery [23]. Especially for treating sudden incidents of water pollution, adsorption method is considered as the first choice, owing to its high efficiency. Therefore, the development of adsorbents using a wide range of raw materials, at low price, and achieving strong adsorption capacity through simple post-adsorption treatment of aniline wastewater has become the focus of research [24].

Polypropylene (PP) is acid- and alkali-resistant, very common material, cheap, and easy to process. After PP is spun by melt-blowing into fibers, it forms a three-dimensional network structure, with smaller mono-filament diameter, higher porosity, and larger specific surface area [25,26,27]. Therefore, it has been widely used as a matrix fiber. However, since PP is non-polar in nature, it does not possess polar and reactive groups along its molecular chains, which limits its ability to adsorb pollutants from wastewaters [28]. Our research group has been actively involved in the preparation of functionalized PP fibers with special properties through grafting, chemical modification, and other methods [29,30,31].

Suspension grafting [32] is a new, environment-friendly, and easy-to-industrialize method for grafting modification. This method usually employs water as the dispersion medium and polymer particles as the dispersed phase. It forms a suspension system, under the action of shear force provided by mechanical stirring. Each suspended polymer particle can be regarded as an independent “reaction bed”. Within this “reaction bed”, the functional monomers undergo graft polymerization under the action of initiators. This polymerization method has advantages of high reaction efficiency, few side reactions, and simple post-processing of products. Li et al. [33] prepared PP-g-PMMA with water as the dispersant and dibenzoyl peroxide as the initiator using the suspension grafting method. The addition of a second monomer, styrene, increased the grafting ratio of monomer to 24.5%. This indicated an effective improvement in the compatibility of PP/ASA mixture.

In this study, the amorphous region of PP was first expanded by quenching treatment, and then functional monomers like acrylic acid (AA) and maleic anhydride (MAH) were grafted onto the surface of PP by suspension grafting. Finally, the PP-g-(AA-MAH) fibers were prepared by melt-blown spinning technology. The adsorption and regeneration performance of PP-g-(AA-MAH) fibers for aniline in aqueous solution were investigated.

## 2. Experimental

### 2.1. Experimental Materials

PP resin especially for the melt-blown process was provided by Shanghai Expert New Material Co. Ltd. (Shanghai, China). Other chemical reagents, such as AA, MAH, xylene, benzoyl peroxide (BPO), acetone, absolute ethanol, sodium hydroxide, hydrochloric acid, etc. were all analytically pure and commercially available.

### 2.2. Preparation of PP-g-(AA-MAH) Fibers

A certain amount of PP resin and xylene were charged into a magnetically stirred reactor and the temperature was raised to 140 °C under stirring. Then, a certain amount of deionized water was rapidly pumped at normal temperature into the reactor to cool down the temperature of the reaction mixture. After this, the temperature of the reactor was maintained at 90 °C and then the mixture was allowed to swell for 30 min. Then, the monomers AA and MAH and BPO initiator were added to the reaction kettle through the high-pressure feed port and graft polymerization was carried out for a certain period of time under strong stirring. The obtained products were washed with deionized water and acetone to remove unreacted monomers and homopolymers. The washed product was dried and then passed through a melt-blown spinning machine [34] to obtain PP-g-(AA-MAH) fibers.

### 2.3. Calculation of Grafting Ratio

PP-g-(AA-MAH) fibers (0.5 g) were placed in a 250 mL round-bottom flask. Xylene (100 mL) and sodium hydroxide-ethanol solution (0.05 mol/L, 30 mL) were added in succession. The mixture was allowed to boil at 120 °C for 30 min. After the solution was cooled to room temperature, 3–5 drops of thymol blue indicator was added and then the mixture was titrated with hydrochloric acid-isopropanol solution (0.05 mol/L) until the blue color just disappeared. The ungrafted PP fibers were used as the blank sample for comparison. The grafting ratio was calculated as follows:

(1) $$G_{p}\%=\frac{\left(V_{0}-V_{1})\times C\times (98.06+72.06\right)}{3\times m}\times 100\%$$

where *G_p_*(%) denotes the grafting ratio; *V*_0_(L) and *V*_1_(L) represent the volumes of HCl-isopropanol solution consumed to titrate PP fibers and PP-g-(AA-MAH) fibers, respectively; *C*(mol/L) denotes the concentration of HCl-isopropanol solution; *m*(g) denotes the mass of the fiber sample; 98.06 g/mol and 72.06 g/mol are the molar masses of MAH and AA, respectively.

### 2.4. Characterization Methods

An X-ray diffractometer (X’TRA, ThermoFisher Scientific, Waltham, MA, USA) was used to characterize the crystal structures of sample before and after quenching. The fluidity and thermal stability of the suspension grafting product were studied by melt indexer (RL-Z1B1, SRD, Shanghai, China) and thermal analyzer (Pyris 1 DSC, PerKinElmer, Waltham, MA, USA), respectively. The functional groups of the modified fibers were analyzed using a Fourier transform infrared spectrometer (Nexus 670, Nicolet, Waltham, MA, USA) and nuclear magnetic resonance spectrometer (DRX500, BRUKER, Karlsruhe, Germany).

### 2.5. Hydrophilicity of PP-g-(AA-MAH) Fiber

The Washburn equation [35] can determine the contact angle of a dynamic liquid by measuring the speed at which the liquid penetrates into the filled powder (Equation (2)). However, the penetration height of wetting liquid is difficult to determine. As the penetration height changes, the air in the capillary tube gets compressed and the air pressure increases [36]. Therefore, Equation (2) can be revised by the relationship between osmotic pressure and height and Equation (3) is obtained.

Let $K_{\theta }=\frac{\beta \gamma_{LV}\cos\theta }{\eta }$, *K_ɵ_* can be obtained from the linear relationship between (Δ*P*)^2^ and *t*.

**Figure 1:**
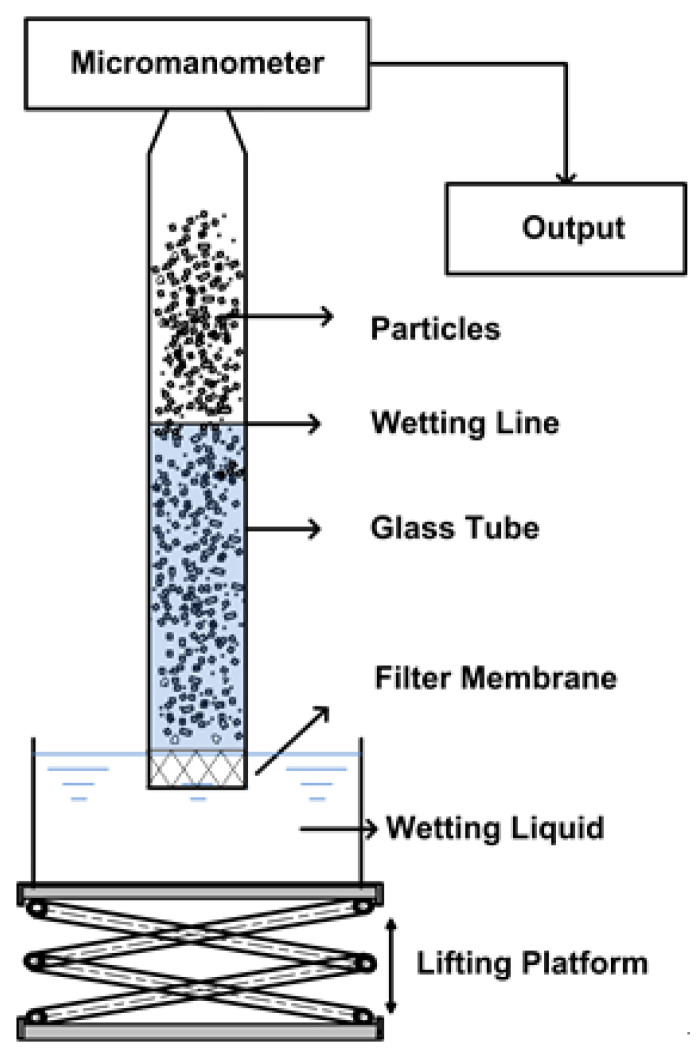
The schematic diagram of the self-made device.

In this study, the water contact angle of degreased cotton with good hydrophilicity was set to 0, that is, when *θ* = 0, then the equation becomes *K*_0_ = $\frac{\beta \gamma_{LV}}{\eta }$. Since the bulk densities of all the test samples was almost the same and deionized water was used as the wetting liquid, the *β*, *η*, and *ϒ_LV_* values of the test samples were approximately the same. Due to these similarities, Equation (4) can be obtained for calculating the water contact angle on the surface of tested fiber. The schematic diagram of the self-made device is shown in Figure 1.

(2) $$h^{2}=\frac{R_{eff\;}\gamma_{LV\;}\cos\theta }{2\eta }t$$

(3) $${\left(\Delta P\right)}^{2}=\frac{\beta \gamma_{LV\;}\cos\theta }{\eta }t$$

(4) $$\theta =\arccos\left(\frac{K_{\theta }\eta }{\beta \gamma_{LV}}\right)=\arccos\left(\frac{K_{\theta }}{K_{0}}\right)$$

where *h* denotes the penetration height of the wetting liquid in time *t*; *R_eff_* denotes the effective capillary radius; *ϒ_LV_* denotes the surface tension of the wetting liquid; *θ* denotes the water contact angle; *η* denotes the viscosity of the wetting liquid; *t* denotes the penetration time; Δ*P* denotes the change in osmotic pressure, and *β* denotes the parameters related to the stacking mode of the stacked bed.

### 2.6. Adsorption and Desorption of Aniline by PP-g-(AA -MAH) Fiber

Ten portions of potassium nitrate (0.01 mol/L, 20 mL) were taken and each portion was transferred to a conical flask. It was purged with N_2_ to remove CO_2_. The pH was adjusted to values between 2–10 using 0.01 mol/L HCl and 0.01 mol/L NaOH solutions. The pH value was recorded as pH_0_. Then, PP-g-(AA-MAH) fibers (0.2 g) was added to the conical flask, flask was sealed, and shaken for 36 h in a water bath at room temperature. Thereafter, the fibers were taken out and the pH value of solution was measured, which was denoted as pH_f_. The pH_0_ values were plotted along the X axis and pH_0_-pH_f_ values along the Y axis, to fit a straight line. The point of intersection of the straight line and the X axis was recorded as the point of zero charge of the PP-g-(AA-MAH) fiber.

Deionized water was used to prepare a mono-contaminated solution containing a certain concentration of aniline and the pH value was adjusted with 0.01 mol/L HCl and 0.01 mol/L NaOH solutions. After adsorption at 25 °C under shaking, the fibers were taken out. After allowing to stand for 5 min, the aniline concentration was determined, and the adsorption capacity was calculated from equation.

(5) $$Q=\frac{(C_{0}-C_{1})V}{m}$$

where *Q* (mg/g) denotes the adsorption capacity; *C*_0_ (mg/L) and *C*_1_ (mg/L) denote the concentrations of aniline in the solution before and after adsorption; *V* (L) denotes the volume of the solution; and *m* (g) is the mass of fiber as absorbent.

The adsorption kinetics were fitted with pseudo-first-order (Equation (6)) and pseudo-second-order (Equation (7)) models.

(6) $$ln(Q_{e}-Q_{t})=lnQ_{e}-K_{1}t$$

(7) $$\frac{t}{Q_{t}}=\frac{1}{K_{2}Q_{e}{}^{2}}+\frac{t}{Q_{e}}$$

where *Q_e_* (mg/g) and *Q_t_* (mg/g) are the amount of adsorbed aniline at equilibrium and at an arbitrary time *t*(min), respectively; and *K*_1_ (1/min) and *K*_2_ (mg/g/min) are the pseudo-first-order and pseudo-second-order rate constants of adsorption, respectively.

The adsorption isotherm was analyzed with the Langmuir and Freundlich models:

(8) $$\frac{C_{e}}{Q_{e}}=\frac{C_{e}}{Q_{m}}+\frac{1}{K_{L}Q_{m}}$$

(9) $$lnQ_{e}=lnK_{F}+\frac{1}{n}lnC_{e}$$

where *C_e_* (mg/L) is the concentration of aniline in solution at equilibrium; *Q_e_* (mg/g) is the equilibrium adsorption capacity; *Q_m_* (mg/g) is the maximum adsorption capacity; *K_L_* (L/mg) is the Langmuir constant; *K_F_* (L/mg) is the Freundlich constant indicative of the adsorption capacity; *n* is a parameter related to the sorption intensity.

The adsorption-saturated PP-g-(AA-MAH) fiber was desorbed by shaking in 0.5 mol/L HCl solution for a certain period of time. The concentrations of aniline in the solution were measured at different times and the desorption ratios were calculated from Equation (10) [37].

(10) $$D=\frac{CV}{mQ}\times 100\%$$

where *D* (%) denotes the desorption ratio; *C* denotes the concentration of aniline in the eluent (mg/L); *Q* is the adsorption capacity of the fiber (mg/g); *V* is the volume of the eluent (L), and *m* denotes mass of fiber (g).

## 3. Results and Discussion

### 3.1. The Mechanism of Suspension Grafting Polymerization of Two Monomers

**Figure 2:**
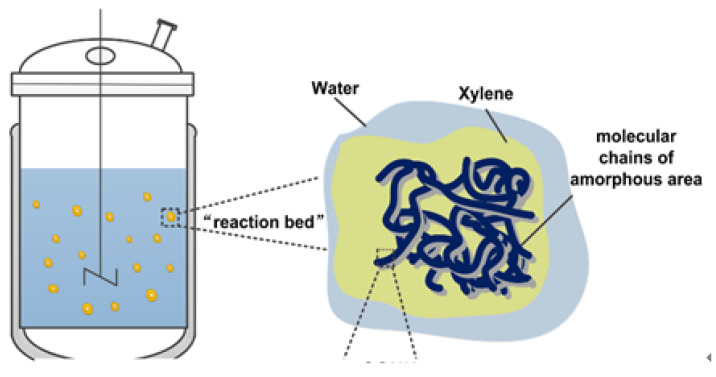
The suspension grafting reaction system.

**Figure 3:**
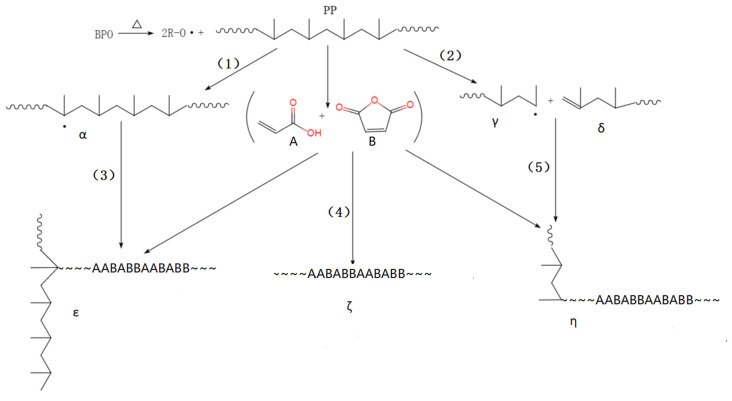
The mechanism of suspension grafting polymerization of AA and MAH monomers.

The schematic diagram of suspension grafting polymerization of AA and MAH monomers [29] is shown in Figure 2 and Figure 3. In the suspension grafting reaction system, under the action of shear force provided by high-speed stirring, each PP resin particle swelled up and was surrounded by xylene. It dispersed into an independent “reaction bed” in deionized water as the dispersant. The grafting polymerization in each “reaction bed” mainly occurred in the amorphous regions of PP resin. In this study, PP resin was quenched before the graft polymerization, in order to enlarge the amorphous region and PP transformed from a resin to a particle with surface micropores. The swelling effect could not only expand the amorphous region further but also enabled the BPO initiator and AA and MAH monomers to diffuse through the surface and into the pores of PP particles more uniformly. Especially, under the action of xylene, they could migrate to the internal micropores of PP more conveniently.

Generally, the initiator first decomposes to form primary free radicals. The primary free radicals can generate PP macromolecular free radicals denoted as α, by capturing α-H from the PP molecular chains (Reaction 1). The macromolecular free radicals, α, react with monomers to form the target product, ε (Reaction 3). However, side reactions like homo-polymerization of monomers (Reaction 4) and PP degradation (Reaction 2), also occur during this process. In the presence of AA, AA first attacks the PP macromolecular radicals α during the grafting polymerization, since AA is an electron provider and has higher reactivity. It can rapidly react with the PP macromolecular radicals α. Moreover, it inhibits side reactions like degradation to form stable PP-AA macromolecular free radicals. The macromolecular free radicals further react with AA or MAH, which is the main reaction pathway. At the same time, due to low reaction temperature, it becomes difficult for the degradation products to react with the monomer (Reaction 5).

### 3.2. Effect of Quenching Treatment

**Figure 4:**
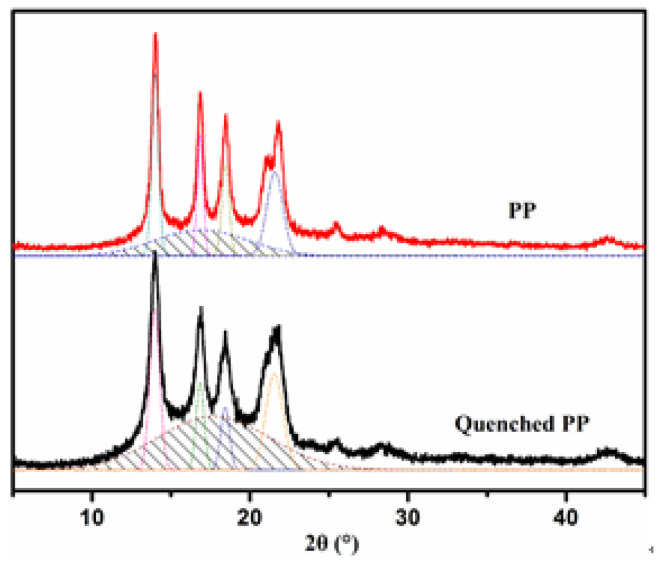
X-ray diffraction patterns of PP before and after the quenching treatment.

The PP used in this research is a special raw material for melt-blown process. It has high crystallinity, but the suspension grafting modification is mainly carried out in the amorphous regions of PP. Therefore, the PP melt can be quenched to reduce its crystallinity, which makes the grafting polymerization easier. It can be seen from the X-ray diffraction patterns of PP before and after the quenching treatment in Figure 4 that the dispersion peak intensity (shaded area in the figure) of the amorphous region of the quenched PP was significantly greater than that of the original PP. Since the quenching process greatly shortens the crystallization time of the PP melt, some of the PP macromolecular chains have no time to move, transform from the disordered coils and then grow into crystals, which results in lower crystallinity.

### 3.3. Effects of Different Parameters on Grafting Ratio in Suspension Grafting Polymerization

**Figure 5:**
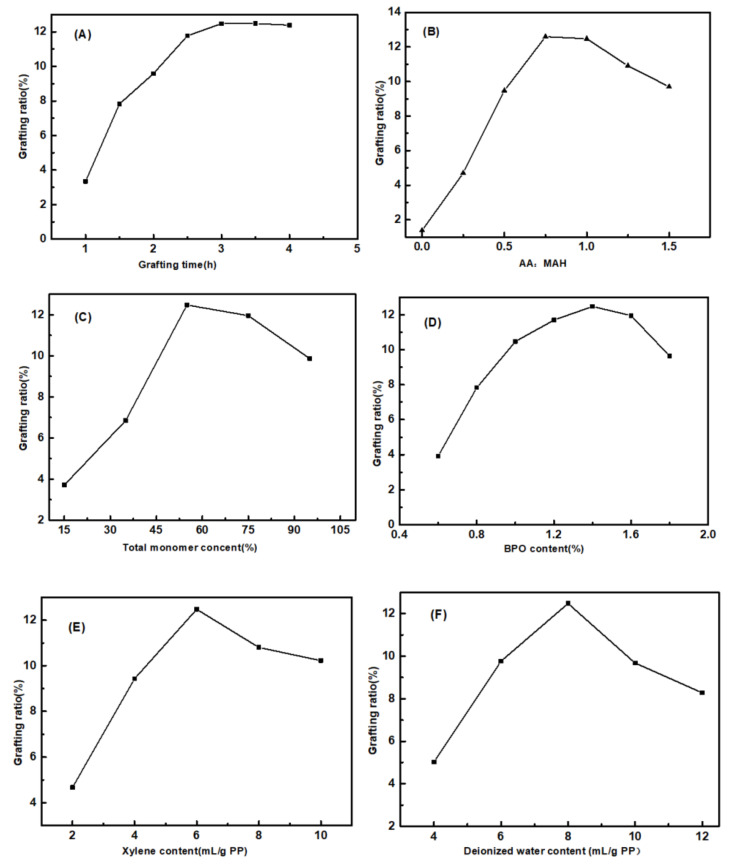
Effects of grafting time (**A**), AA/MAH ratio (**B**), total monomer content (**C**), and the dosages of BPO (**D**), xylene (**E**), and deionized water (**F**) on grafting ratio.

The main factors affecting grafting in suspension grafting polymerization include the grafting time, the AA/MAH ratio, the total amount of monomers, and the dosages of BPO, xylene, and deionized water. Therefore, effects of the six factors on the grafting ratio were all investigated and the results are shown in Figure 5.

It can be seen from Figure 5A that the grafting ratio increased rapidly with increase in grafting time and it reached a stable state after three hours. The half-life period of the initiator BPO at 90 °C is 1 h [29]. In the initial stages of the reaction, BPO decomposed into a large number of primary free radicals, which rapidly initiated the grafting reaction. In addition, the monomer concentration was also high at this time, due to which the grafting ratio increased rapidly. After 3 h, BPO was basically decomposed and the monomer concentration was also lower, exhibiting a more stable grafting ratio.

From Figure 5B, it is evident that when the mass fraction of AA was 0, the grafting ratio was only 1.38%. Subsequently, as the AA/MAH ratio increased, the grafting ratio increased at first and then decreased. With increase in the amount of monomer AA added, the concentration of macromolecular free radicals with AA chains increased. This increased the probability of free radicals contacting with PP. In addition, the monomer MAH could not undergo self-polymerization, due to its steric hindrance. When AA was added, it could form a copolymer with MAH and then graft with the PP segment [38], due to which the grafting ratio increased. However, as the amount of AA added continued to increase, the probability of capturing primary free radicals increased, thereby forming a greater number of AA-containing macromolecular free radicals. The probability of termination due to recombination between these free radicals increased, which resulted in lower grafting ratio. In addition, more the number of AA molecules, more was the probability of AA initiation, which then reduced the probability of formation of macromolecular free radicals of PP, thereby affecting the grafting ratio.

It can be seen from Figure 5C that as the total amount of monomers AA and MAH increased, the grafting ratio increased at first and then decreased. Higher monomer concentration increased the effective collisions between the monomers and PP macromolecular radicals, thereby increasing the grafting ratio. However, grafting polymerization mainly occurred in the amorphous regions of PP. Although the amorphous regions of PP expanded after quenching, the reaction space was still limited. Too high monomer concentration led to more homopolymerization as the side reaction. Additionally, the mass transfer of monomer and initiator were also affected, so the grafting ratio decreased.

With an increase in the amount of initiator BPO, the grafting ratio first increased and then decreased. When the amount of added BPO accounted for 1.4% of PP, the grafting ratio reached the maximum value of 12.47% (Figure 5D). On heating, BPO first decomposed into benzoyl radicals. Due to steric hindrance, benzoyl radicals were further decomposed into benzene radicals with stronger capability for H atom. Benzene radicals flowed and diffused slowly in the PP particles within limited space, depriving the α-H from the PP chains to form PP macromolecular radicals and initiating monomer polymerization. Therefore, by appropriately increasing the amount of BPO, a greater number of benzene radicals could be generated. This increased the number of monomers participating in the grafting reaction and increased the grafting ratio. However, excessively high concentration of benzene radicals could cause explosive polymerization and lead to an increase in homopolymerization side reactions. This would again affect the progress of the grafting reaction and also result in degradation of PP [39].

It can be seen from Figure 5E that, with the increase in amount of xylene, the grafting ratio increased rapidly and then decreased slightly. With xylene concentration of 6 mL/g PP, the grafting ratio reached the maximum value of 12.47%. The monomers used in this study possessed polar groups, and hence their solubility parameters were quite different from that of PP, which implied lower swelling capability of PP. The addition of certain amount of xylene helped to increase the swelling degree of PP and provided greater space for the grafting reaction. Meanwhile, the addition of xylene also kept the concentration of water-soluble monomers at the interface to a low level. This effectively reduced homo-polymerization as side reaction and increased the grafting ratio. However, too much xylene readily formed a film covering the surface of PP, which hindered the grafting reaction between monomer and PP and affected the grafting ratio.

Figure 5F shows that as the amount of deionized water as dispersant increased, the grafting ratio increased rapidly and then decreased. As the amount of deionized water reached 8 mL/g PP, the grafting ratio reached the maximum value. If the amount of deionized water added was too less, the dispersion of PP into multiple independent “reaction beds” became difficult, since PP particles agglomerated in the reactor to form agglomerates, resulting in a low grafting ratio. With too much of deionized water, although the PP particles could form numerous independent “reaction beds”, the concentration of monomer dissolved in the water decreased. Hence, the monomer concentration gradient between the dispersion medium and the interfacial reaction layer became smaller. As a result, it was difficult for the monomer molecules to enter the interfacial layer and participate in the grafting polymerization reaction, due to which the grafting ratio decreased.

Based on the aforementioned study of the effect of parameters of suspension graft polymerization on the grafting rate, the optimal combination of parameters for suspension graft polymerization are presented in Table 1.

### 3.4. Effect of Suspension Grafting on the Performance of Melt-Blown Spinning Product

**Figure 6:**
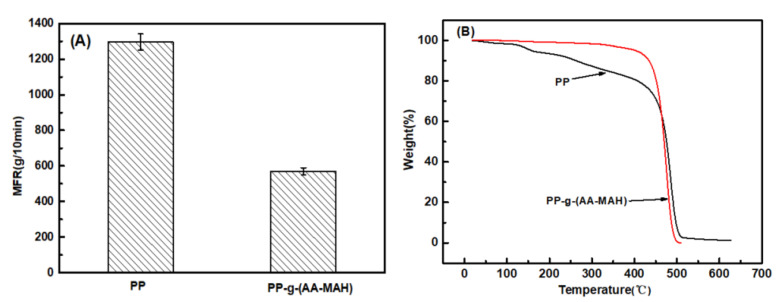
MFR (**A**) and TG (**B**) of PP and PP-g-(AA-MAH) fibers.

Melt-blown spinning has strict requirements of fluidity and thermal stability of raw materials. PP resin itself has greater fluidity, but after its modification with polar groups, the interactions between molecular chains increase and the fluidity is affected. In addition, in the melt-blown spinning process, the raw materials are melted at high temperature and extruded through the screw. Hence, only better thermal stability can ensure that no decomposition of the raw materials occurs during the process. Therefore, the melt flow rate (MFR) and thermogravimetric (TG) of PP and PP-g-(AA-MAH) were investigated to determine whether it can be spun into fibers. The results are presented in Figure 6.

It can be seen from Figure 6A that MFR (570 g/10 min) of the product PP-g-(AA-MAH) of suspension grafting was lower than that of the PP raw material (1296 g/10 min), implying decrease in fluidity. However, the MFR was still higher than that required for the melt-blown process of PP melt (400 g/10 min). The decrease in MFR could be attributed mainly to the strengthening of hydrogen bonds between the grafted polar chains [29,34]. Compared with the TG curves of unmodified PP in Figure 6B, the product of suspension grafting PP-g-(AA-MAH) had better thermal stability. PP-g-(AA-MAH) showed no significant weight loss before 250 °C, and hence the melt-blown spinning at maximum temperature of 220 °C basically did not cause decomposition.

### 3.5. Characterization of Fibers

#### 3.5.1. Fourier Transform Infrared Analysis (FT-IR)

**Figure 7:**
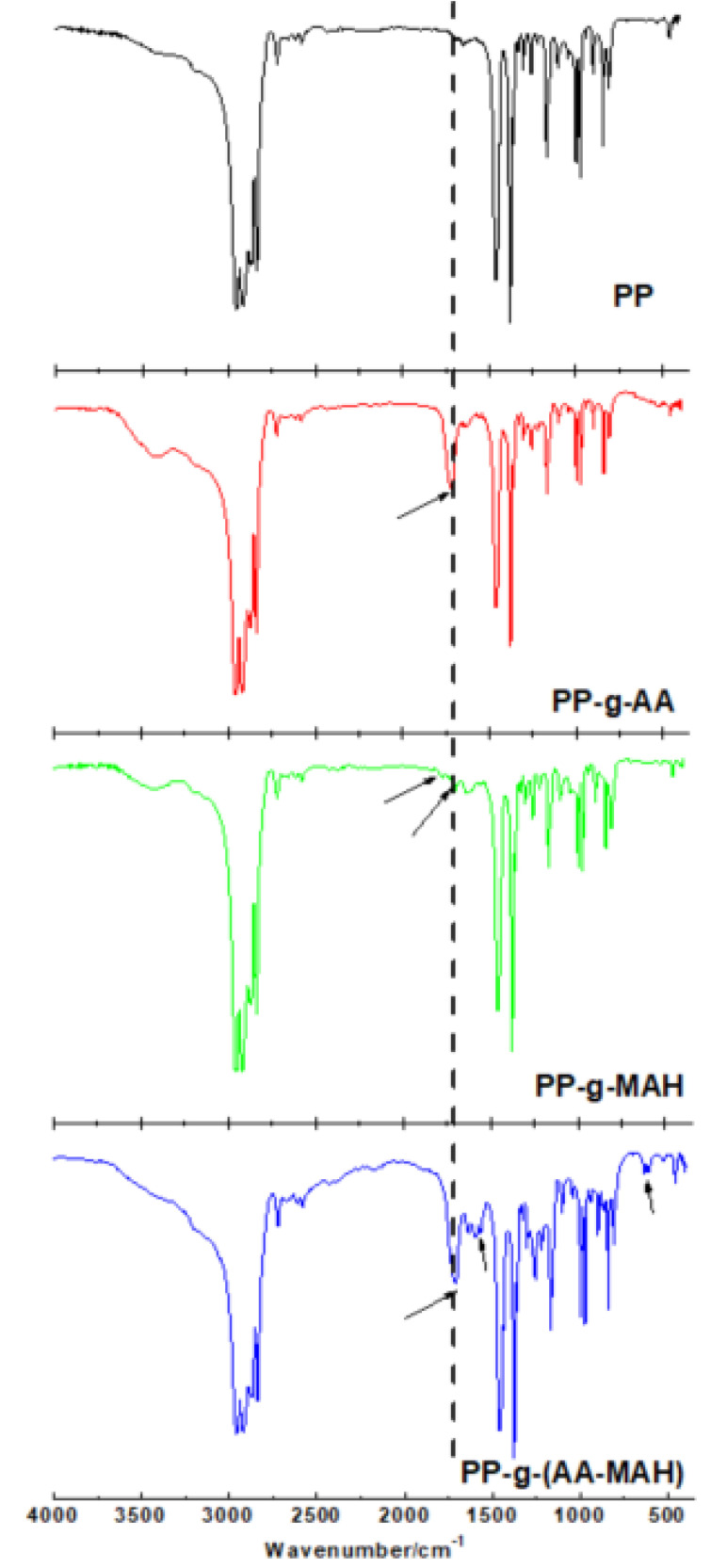
FTIR spectra of PP, PP-g-AA, PP-g-MAH and PP-g-(AA-MAH) fibers.

PP and its products of suspension grafting - PP-g-AA, PP-g-MAH, and PP-g-(AA-MAH) were spun by melt-blowing and processed into fibers. Their FTIR spectra (the total monomer contents were the same during the suspension grafting process) are shown in Figure 7. The fibers of modified PP-g-AA, PP-g-MAH, and PP-g-(AA-MAH) showed unique characteristic absorption peaks of PP, viz. symmetrical (2924 cm^−1^) and asymmetrical (2842 cm^−1^) C-H stretching vibrations of –CH_3_, the flexural vibrations of –CH_2_- at 1380 cm^−1^, and absorption of C–H at 1450 cm^−1^. Compared with that of PP fiber, the FTIR spectrum of PP-g-AA fiber showed peak for C=O stretching vibrations of the carboxyl group (1720 cm^−1^), indicating the successful grafting of AA onto the PP chain. Compared to the spectrum of PP fiber, PP-g-MAH showed a peak at 1780 cm^−1^, which was attributed to the symmetric and asymmetric vibrations of C=O of acid anhydride. The peak at 1720 cm^−1^ was ascribed to the C=O of carboxylic acid, formed by the hydrolysis of anhydride. The FTIR spectra of PP-g-(AA-MAH), PP-g-AA, and PP-g-MAH fibers all showed peaks for stretching vibrations of C=O of the carboxyl group at 1720 cm^−1^. The difference was that the FTIR spectrum of PP-g-(AA-MAH) had stronger intensity for C=O peak, indicating that its carboxyl content was higher than those of PP-g-AA and PP-g-MAH fibers. However, the FTIR spectrum of PP-g-(AA-MAH) fiber did not show peaks for symmetrical and asymmetrical vibrations of C=O of acid anhydride in the PP-g-MAH spectrum. This was due to the fact that MAH was grafted on to PP in the form of maleic acid [39,40]. FTIR results confirmed the successful grafting of AA and MAH onto PP fibers.

#### 3.5.2. ^1^H-NMR Analysis

**Figure 8:**
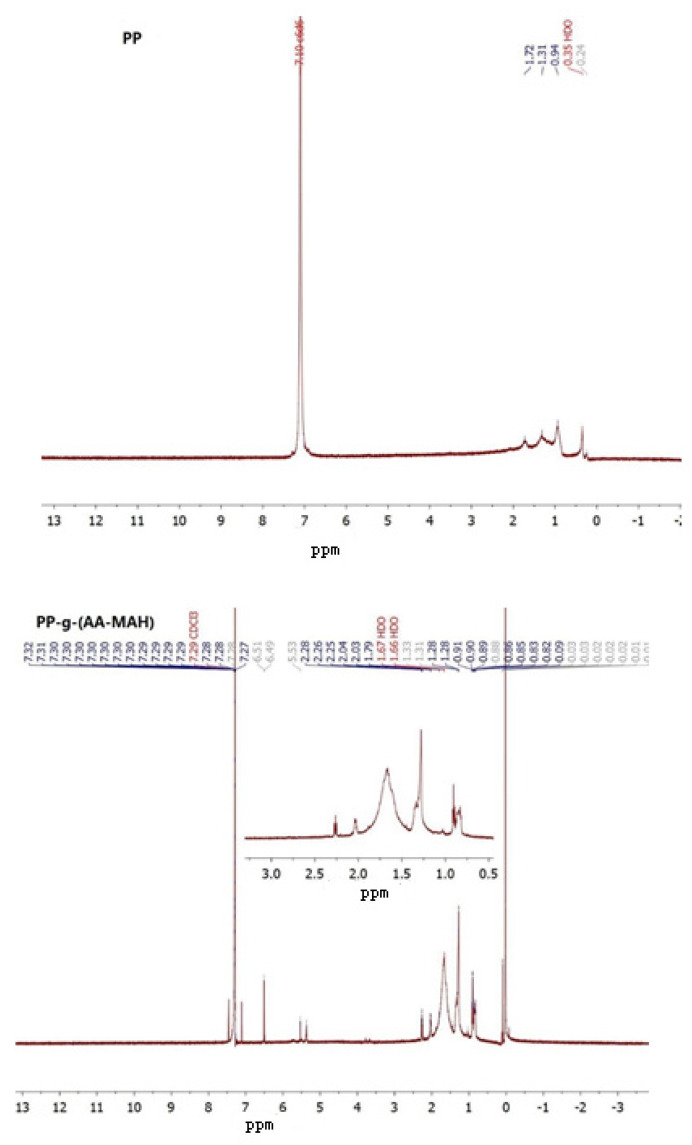
^1^H-NMR analysis of PP and PP-g-(AA-MAH) fibers.

The carboxyl group in PP-g-(AA-MAH) fiber was linked to –CH–. From the ^1^H-NMR analysis (Figure 8), it was evident that compared with PP fiber, PP-g-(AA- MAH) fiber showed a chemical shift at around 2.3 ppm [41], indicating that the carboxyl groups were successfully grafted onto the surface of PP fibers.

#### 3.5.3. Analysis on Hydrophilicity

**Figure 9:**
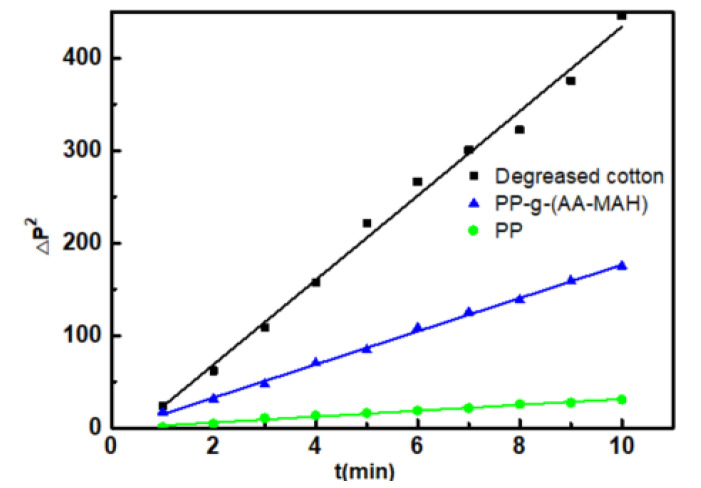
Linear fitting relationship of (Δ*P*)^2^ of degreased cotton, PP-g-(AA-MAH) and PP fiber with t.

Figure 9 showed a linear fitting relationship of (Δ*P*)^2^ of degreased cotton, PP-g-(AA-MAH) fiber and PP fiber with *t*. It could be seen from the figure that the *K*_0_ of degreased cotton was 45.66, whereas *K_ɵ_* of PP-g-(AA-MAH) fiber and PP fiber were 17.95 and 3.19, respectively. According to Equation (4), the calculated water contact angles of PP-g-(AA-MAH) fiber and PP fiber were 66.85° and 85.98°, respectively, which indicated increased hydrophilicity of PP-g-(AA-MAH) fibers.

### 3.6. Investigation of Aniline-Adsorption Performance of PP-g-(AA-MAH) Fiber before and after Regeneration

#### 3.6.1. Effect of pH on the Adsorption Capacity

**Figure 10:**
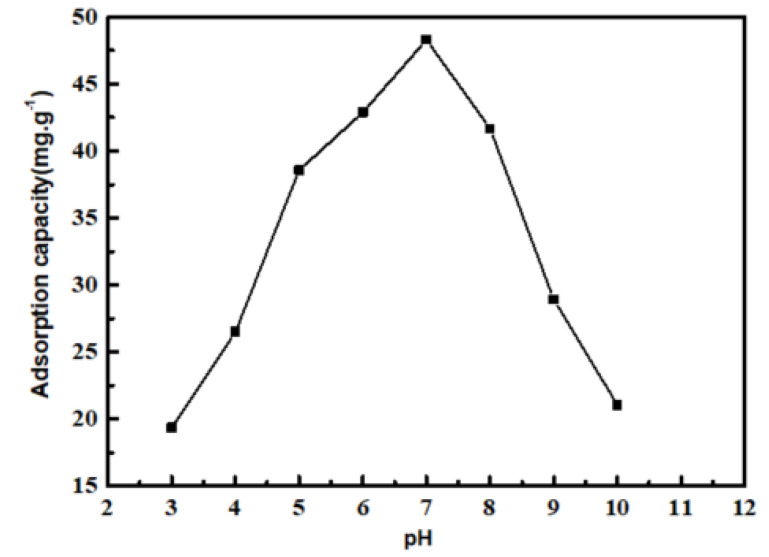
The effect of the pH value on the adsorption capacity.

Solution pH value influences the surface properties of PP-g-(AA-MAH) fibers and the state of aniline in water, which in turn has an effect on the adsorption. Figure 10 shows the effect of pH value on the adsorption capacity of PP-g-(AA-MAH) fibers. The adsorption capacity first increased and then decreased in the pH range from 3 to 10. The adsorption capacity reached the maximum value of 48.3 mg/g, when the solution was neutral.

**Figure 11:**
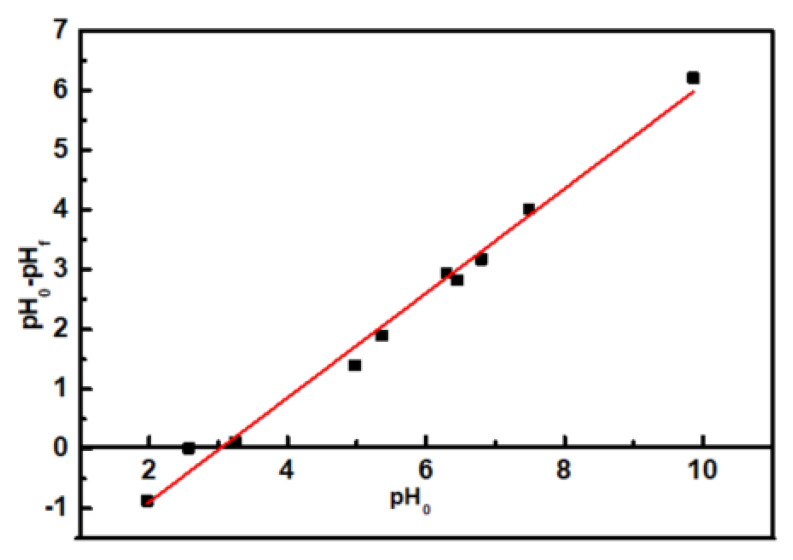
The point of zero charge of PP-g-(AA-MAH) fiber in the aqueous solution.

Aniline is weakly alkaline and has pK_b_ value (dissociation constant) of 9.4. In other words, aniline exists as C_6_H_5_NH_3_^+^ below 9.4 pH and in molecular form above 9.4 pH [20,21]. Combining this with the point of zero charge (3.02) of the PP-g-(AA-MAH) fiber in the aqueous solution (from Figure 11), the surface of PP-g-(AA-MAH) fiber was protonated and positively charged below 3.02. Therefore, the electrostatic repulsion between aniline (in the form of C_6_H_5_NH_3_^+^) and positively charged surface led to its poor adsorption. Above 3.02, the surface of PP-g-(AA-MAH) fiber was negatively charged due to deprotonation. The number of –COO^−^ groups on the surface increased and the electrostatic attraction was strengthened. Hence, the adsorption capacity of PP-g-(AA-MAH) fibers towards aniline gradually increased between pH 3~7. As pH value continued to rise and the aqueous solution turned alkaline, the reduction of C_6_H_5_NH_3_^+^ led to the reductions in adsorption capacity, although the number of –COO^−^ groups increased. Above pH 9.4, aniline mainly existed in the molecular form and had lower solubility, which was not conducive for adsorption, and hence the adsorption capacity decreased further.

#### 3.6.2. Adsorption Kinetics

**Figure 12:**
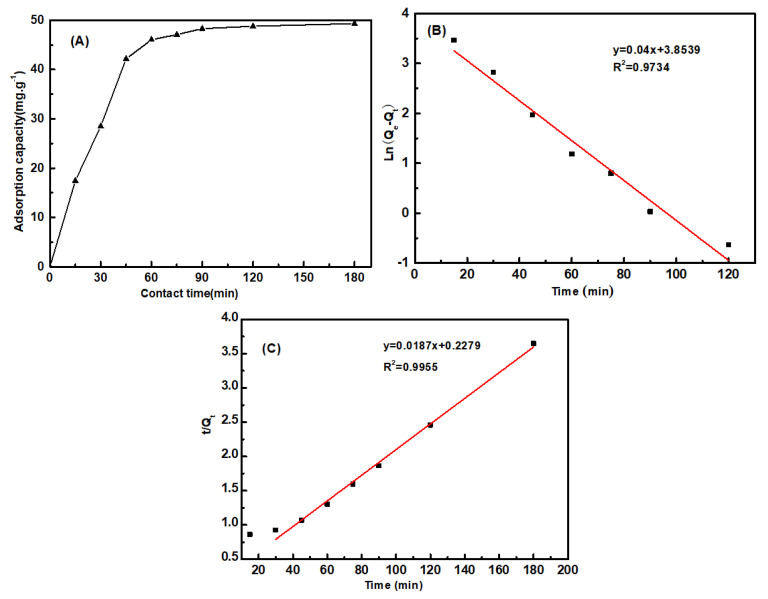
The effect of contact time on adsorption capacity (**A**) and the linearized pseudo-first-order (**B**) and pseudo-second-order (**C**) kinetics.

Under the conditions of 25 °C, pH = 7, and initial aniline concentration of 300 mg/L, the adsorptivities of aniline on PP-g-(AA-MAH) fibers at different adsorption times were investigated, and the kinetics was studied, as shown in Figure 12. Figure 12A showed that PP-g-(AA-MAH) fibers had faster adsorptivity for aniline within the first 45 min, with adsorption capacity reaching up to 42.2 mg/g. Then, the adsorption capacity increased slowly until it reached saturation as 48.3 mg/g at 120 min. The faster rate of adsorption within the first 45 min was the result of the large number of active sites on the surface of PP-g-(AA -MAH) fibers. With the prolongation of adsorption time, the number of active sites on the surface of PP-g-(AA-MAH) fibers gradually decreased along with decrease in aniline concentration. As a result, the driving force for adsorption obviously weakened and the adsorption capacity increased slowly until it reached saturated adsorption. From the linear fitting results (Figure 12B,C), The R^2^ of the pseudo-first-order kinetic model is 0.9734, while for the pseudo-second-order kinetic model, R^2^ is 0.9955. Hence, the adsorption for aniline is better fitted by the pseudo-second-order kinetic model, indicating that the adsorption is controlled by chemical phenomena [42]. Hence, the electrostatic attraction is the main driving force for aniline adsorption on PP-g-(AA -MAH) fibers.

#### 3.6.3. Adsorption Isotherm

**Figure 13:**
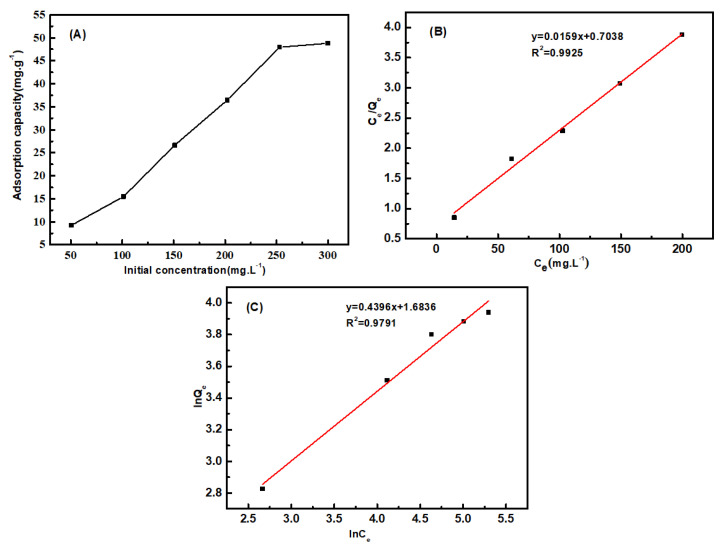
The effect of initial aniline concentration on adsorption capacity (**A**) and the linearized Langmuir(**B**) and Freundlich (**C**) models.

The adsorption isotherm was drawn based on the results obtained using different initial aniline concentrations. As can be seen from Figure 13A, the adsorption capacity increased with the increase in initial aniline concentration, and eventually became stable. With the increase in aniline concentration, the probability of collision between functional groups on fibers and aniline increased, and so the adsorption capacity increased. However, the number of functional groups was limited, and the adsorption capacity eventually tended to be stable. The linearized versions of the two models are illustrated in Figure 13B,C and the values of the isotherm parameters are listed in Table 2. It was found that the R^2^ value of Langmuir and Freundlich was 0.9925 and 0.9791, respectively, and the adsorption for aniline of PP-g-(AA-MAH) fibers is better fitted by the Langmuir model. The uptake of aniline occurs on the homogeneous surface of PP-g-(AA-MAH) fibers by monolayer adsorption. The maximum theoretical adsorption capacity for aniline of PP-g-(AA-MAH) fibers is 62.7 mg/g.

#### 3.6.4. Comparison of Adsorption Capacity with other Adsorbents

Comparisons between maximum adsorption capacities (q_max_) of PP-g-(AA-MAH) fibers and other adsorbents for aniline reported in the literature are presented in Table 3. The results show that PP-g-(AA-MAH) fibers exhibits a reasonable capacity for aniline adsorption.

#### 3.6.5. Desorption and Regeneration

**Figure 14:**
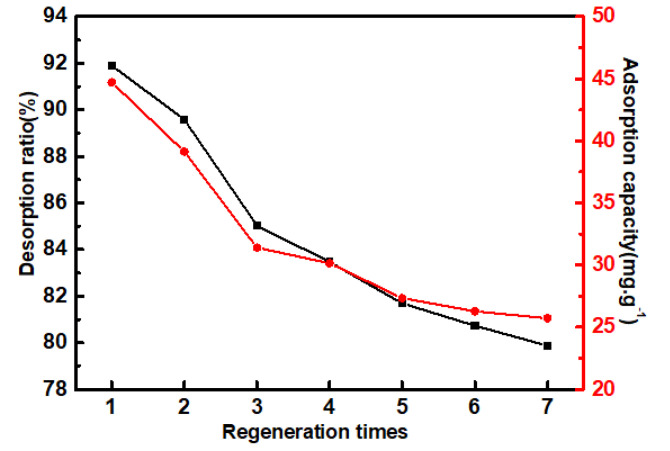
The desorption ratio and adsorption capacity of regenerated fiber.

The regeneration performance of PP-g-(AA-MAH) fibers was investigated using the desorption ratio and adsorption capacity of regenerated fiber, as shown in Figure 14. It can be seen that after seven adsorption-desorption cycles, the desorption ratio of the PP-g-(AA-MAH) fiber still remained above 80% and the adsorption capacity for aniline could still reach 25.72 mg/g, indicating that PP-g-(AA-MAH) fiber had good regeneration–adsorption performance.

## 4. Conclusions

With PP as the matrix, AA and MAH as functional monomers, PP-g-(AA-MAH) fiber was prepared by suspension grafting and melt-blown spinning technology. The technology can be easily scaled-up to industrial level and the fibers can be used to adsorb aniline. The maximum grafting ratio was 12.47% under the following optimal suspension grafting conditions: grafting time of 3 h, AA: MAH = 0.75, total monomer content of 55%, BPO content of 1.4%, xylene content of 6 mL/g PP, and deionized water content of 8 mL/g PP. Melt flow-rate and thermal analysis indicated that the suspension grafting product had good fluidity and thermal stability, and could be spun by melt-blown process. FTIR and NMR analyses showed that AA and MAH were successfully grafted onto PP fibers. The hydrophilicity of PP-g-(AA-MAH) fiber was enhanced. Therefore, the adsorption for aniline from aqueous solution was fast and the adsorption capacity could reach up to 42.2 mg/g at 45 min and pH = 7. The kinetics of adsorption followed the pseudo-second-order model and the adsorption isotherm data were better fitted by the Langmuir model. Moreover, the fibers had good regeneration performance.

## Figures and Tables

**Table 1:** The optimal combination of parameters for suspension graft polymerization.

PP	Monomers		Xylene	Deionized Water	BPO	Grafting Time	Grafting Ratio
	AA	MAH					
200 g	47g	63g	1.2L	1.6L	2.8g	3h	12.47%

**Table 2:** Isotherm parameters.

Langmuir			Freundlich		
Q_m_/mg·g^−1^	K_L_	R^2^	n	K_F_/mg·g^−1^	R^2^
62.7	0.0226	0.9925	2.2745	5.3851	0.9791

**Table 3:** Adsorption capacity of various adsorbents for aniline

Adsorbents	q_max_/mg·g^−1^	Reference
Modified pine sawdust	21.8	[43]
Activated carbon/chitosan composite	22.9	[44]
PAM/SiO_2_	52.0	[45]
Modied ATP	16.1	[46]
Cr-bentonite	21.6	[47]
Modified jute fiber	125	[20]
Edible fungus residue activated carbon	27.1	[21]
PP-g-(AA-MAH) fibers	48.3	This work
